# Research on highway traffic flow prediction model and decision-making method

**DOI:** 10.1038/s41598-022-24469-y

**Published:** 2022-11-19

**Authors:** Yuyu Zhu, QingE Wu, Na Xiao

**Affiliations:** 1 Faculty of Engineering, Huanghe University of Science and Technology, Zhengzhou, 450000 China; 2 School of Electrical and Information Engineering, Zhengzhou University of Light Industry, Zhengzhou, 450002 China

**Keywords:** Engineering, Mathematics and computing

## Abstract

In order to solve the problem of traffic congestion in a certain area, this paper develops a set of traffic optimization decision system. For analyzing the actual traffic conditions and calculating the traffic volume, density and traffic speed, a traffic prediction model is established and updated iteratively to modify the prediction model parameters. Based on this model, the congestion degree is estimated at the current road section, thus, an intelligent decision-making and the coordinated optimization methods are proposed. Moreover, this paper implements some application experiments on the isometric road of a three-intersection and obtains better prediction results of traffic density and traffic speed on the three-section highway. At the same time, compared with other existing prediction methods, the prediction model presented in this paper not only has higher accuracy, shorter prediction time and stronger anti-interference ability, but also has better effect on vehicle diversion. In addition, it also greatly relieves the traffic pressure on the road, maximizes the complementary advantages between intersections, and balances the good cooperation between each intersection.

## Introduction

With the rapid development of economy and society, urban traffic congestion has become increasingly serious, For coordinating networked vehicles and traffic lights at urban intersections, Rodriguez et al.^1^ proposed a decentralized control framework. However, the framework didn’t discuss the different gradient to dynamically coordinate the vehicle's planned intersection arrival time. For better highway event detection, traffic analysis and traffic forecast,^2^ proposed a method that could reduce delays by 55–70 percent in different traffic conditions. However, the highway traffic flow model was actually a high-order nonlinear time-varying system, which makes the state estimation of the model a very difficult problem. In order to achieve accurate estimation of traffic flow, a traffic prediction optimization model system is proposed in this paper.

In fact, in order to solve the problem of traffic congestion and congestion, the main method of some studies^3–5^ was to establish mathematical models for traffic flow to control and optimize the entire traffic system. For solving some problems of urban traffic control and management, Jiahui et al.^6^ proposed a dynamic and deadline-oriented road pricing mechanism model to open up a new solution to the management and control of traffic. Although these studies had advanced methods, the effect of control optimization was not satisfactory due to the randomness and complexity of the traffic system, especially in some megacities where road congestion and traffic accidents occur frequently. For optimized design of some technical parameters, Junwei et al.^7–9^ established the traffic flow cellular automata model based on the construction strength, anti-collision ability and mechanical properties of expressway traffic safety facilities. However, these studies did not solve a real complex various system and the limitations of the traffic flow model.

For a good optimization on construction parameters, Qiu et al.^10^ proposed a construction quality control method for expressway traffic safety facilities based on parameter fusion tracking control and piecewise linear regression analysis of construction schedule, but the mode cannot meet the needs of urban traffic. In addition, Kumar et al.^11^ proposed an effective traffic control system based on ant colony algorithm and Internet of vehicles technology, but this management method is not suitable for all industries. To avoid traffic congestion, Sun Danying^12^ proposed an intellectual administrative system of traffic events. However, it turned out that the system could not entirely ensure the smooth road conditions and safe driving. Kuppusamy et al.^13^ proposed a novel intelligent traffic control framework, but it was not effective for complex congestion. For large and complex traffic conditions, Wang et al.^14^ put forwards a more accurate trunk line control system based on the concepts of red wave and green wave, however, the traffic pressure and relieve congestion reached only 70% at the intersection. For most advanced traffic simulation and evaluation, Louati et al.^15^ developed Pannal, which could effectively solve the problem of traffic congestion and facilitate the emergency vehicles. At the same time, Yang et al.^16^ proposed an improved near-end policy optimization algorithm, but they ignored the relationship between vehicles and traffic lights, so the effect of urban traffic control is not comprehensive enough. For traffic signal control, Huang Mingxia et al.^17^ proposed an intelligent traffic system based on FPGA traffic signal light, and Tajdari et al.^18^ proposed a new adaptive control method based on the reference of unknown time-varying basic graph model. However, the previous studies did not give a solving method for high order nonlinear model of traffic congestion.

**Figure 1:**
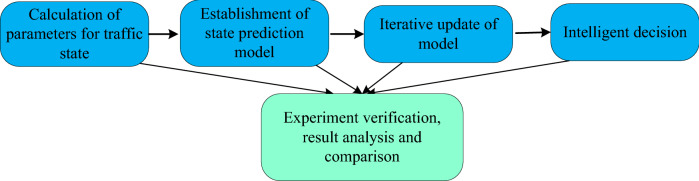
Flow chart of traffic flow prediction and decision method.

To solve the high order nonlinear model of traffic congestion, this paper proposes the model linearization iterative updating method and develops a traffic prediction and decision system. The system consists of four parts: (1) An analysis of actual traffic conditions, that is, calculation of traffic volume, density and speed; (2) Establishment of traffic flow state prediction model, that is, congestion degree, influence of future time period on surrounding road branches, and state estimation modeling; (3) Iterative update of model, that is, in the application of traffic flow analysis, studying the traffic flow data under various conditions and modifying the parameters of the prediction model until the requirements are met; (4) Intelligent decision-making, that is, game algorithm in regional roads, traffic optimization, regional scheduling, diversion of traffic congestion and smooth roads. Figure 1 shows the framework of proposed methods and system construction. These studies provide theoretical ideas and decision-making support for government construction, urban planning and road length and width design, as well as target detection and tracking methods for places and departments using video stream.

Article highlights:

Simultaneously, the novelty of present work had been abstracted as follows:

- A traffic coordination optimization method is proposed.
- A traffic flow prediction model is established.
- An intelligent decision-making method is given, and a coordinated optimization method for regional traffic is also given.
- A set of traffic optimization decision system is developed.

## Calculation of parameters on traffic flow condition

According to works of Payne and Papa Georgiou et al.^1,2^, the model of traffic flow is discussed as follows:

Take an interval segment with intersection in a region for example. Divide the segment appropriately into $L$ segments, each of which $\Delta_{i}$ is spatial sampling length. Traffic detectors are set at the beginning and end of the whole road to provide measured data as the input of the dynamic model, including traffic flow $q_{i} \left( k \right)$ and average speed $v_{i} \left( k \right)$. The detection period $T$ is 60 s, usually a cycle of 10–60 s. The density of traffic flow $\rho_{i} \left( k \right)$ is calculated from the traffic flow $q_{i} \left( k \right)$ and average speed $v_{i} \left( k \right)$, and these parameters based on^1,2^ are calculated as follows:

1 $$ q_{i} \left( k \right) = \alpha \rho_{i} \left( k \right)v_{i} \left( k \right) + \left( {1 - \alpha } \right)\rho_{i + 1} \left( k \right)v_{i + 1} \left( k \right) $$

2 $$ \rho_{i} \left( {k + 1} \right) = \rho_{i} \left( k \right) + \frac{T}{{\Delta_{i} }}\left[ {q_{i - 1} \left( k \right) - q_{i} \left( k \right) + r_{i} \left( k \right) - s_{i} \left( k \right)} \right] $$

3 $$ \begin{aligned} v_{i} \left( {k + 1} \right) & = v_{i} \left( k \right) + \frac{T}{\tau }\left\{ {v\left[ {\rho_{i} \left( k \right)} \right] - v_{i} \left( k \right)} \right\} \\ & \;\;\; + \frac{T\xi }{{\Delta_{i} }}v_{i} \left( k \right)\left[ {v_{i - 1} \left( k \right) - v_{i} \left( k \right)} \right] - \frac{\gamma T}{{\tau \Delta_{i} }}\frac{{\rho_{i + 1} \left( k \right) - \rho_{i} \left( k \right)}}{{\rho_{i} \left( k \right) + \lambda }} \\ \end{aligned} $$

where

4 $$ v\left( \rho \right) = v_{f} \exp \left[ { - \left( {{1 \mathord{\left/ {\vphantom {1 b}} \right. \kern-\nulldelimiterspace} b}} \right)\left( {{\rho \mathord{\left/ {\vphantom {\rho {\rho_{cr} }}} \right. \kern-\nulldelimiterspace} {\rho_{cr} }}} \right)^{b} } \right] $$

In addition, in the formula, $r_{i} \left( k \right)$ is the traffic flow rate in the $i$th paragraph at the entrance at time $kT$, and $s_{i} \left( k \right)$ is the traffic flow rate at the exit. The parameter $v_{f}$ represents the free speed of traffic flow, namely, the maximum speed of current traffic flow. $\rho_{cr}$ is the density of traffic flow when the traffic flow reaches the maximum, namely, the critical density, and $b,\tau ,\gamma ,\xi ,\lambda ,\alpha$ is the adjustment coefficient of the equation. Equation (4) is a steady-state traffic flow model, which describes the velocity density relationship of steady-state traffic flow.

## Establishment of prediction model

According to the analysis of high-speed traffic flow data in various situations, these measurement data can be established. The dynamic model is carried out multiple tests by simulation and experiments based on the above calculation of the parameters of the traffic flow dynamic model. Moreover, according to the principle of automatic control nonlinear system, the state of traffic flow and its measurement values can be obtained at the $k$ time:

5 $$ x\left( {k + 1} \right) = f\left[ {x\left( k \right)} \right] + \Gamma \left[ {x\left( k \right)} \right]w\left( k \right) $$

6 $$ y\left( {k + 1} \right) = h\left[ {x\left( {k + 1} \right)} \right] + v\left( {k + 1} \right) $$

where $w(k)$ and $v(k)$ are the zero-mean noise vectors, and

$$E[w(k)] = 0,\quad E[w(k)w^{\prime}(j)] = Q(k)\delta_{kj}, \quad  E[v(k)] = 0,\quad E[v(k)v^{\prime}(j)] = S(k)\delta_{kj}.$$

$Q(k)$ is a variance matrix of the system noise, $S(k)$ a measure noise matrix, $\Gamma \left[ {x(k)} \right]$ is the noise drive matrix, which these can be obtained by experiments and measures. $f\left[ {x\left( k \right)} \right]$ and $h\left[ {x\left( k \right)} \right]$ are two mapping function of state and measure mapping function to perform the state estimation and measure value, respectively. According to the calculation of positive definite matrix, $f\left[ {x\left( k \right)} \right]$ and $h\left[ {x\left( k \right)} \right]$ are given by repeated experiments and reasoning as follows:

7 $$ f\left[ {x\left( k \right)} \right] = ce^{{ - \frac{1}{2}\sum\limits_{i = 1}^{M} {\left( {y\left( k \right) - x\left( k \right)} \right)^{T} Q^{ - 1} \left( k \right)\left( {y\left( k \right) - x\left( k \right)} \right)} }} $$

8 $$ h\left[ {x\left( k \right)} \right] = \sum\limits_{i = 1}^{M} {\frac{{S^{2} (k)}}{{S^{2} (k) + \left( {y\left( k \right) - x\left( k \right)} \right)^{T} Q^{ - 1} \left( k \right)\left( {y\left( k \right) - x\left( k \right)} \right)}}} $$

where $c$ is an adjustment constant, and $M$ is the measurement number of sensors.

To define a following norm on distance, if there is a certain state value $\hat{x}\left( k \right)$ meet to the following formula for the corresponding measurement value $y(k)$, $\hat{x}\left( k \right)$ is the established state of traffic flow at the $k$ time.

9 $$ \left\| {\hat{x}\left( {k + 1} \right) - y\left( {k + 1} \right)} \right\| < \delta $$

where $\delta$ is a given threshold.

## Iterative update of prediction model

In order to make the developed system predict traffic flow state more accurately, a new traffic prediction method, called iteration, is presented here. The iterative update algorithm (IUA) needs to integrate information of different state models, and update state, state error, measurement error, process gain value $K\left( {k + 1} \right)$ and covariance accordingly $P\left( {k + 1} \right)$.

### Iterative update of model

Further, according to the above formula (5)–(9), the state value $\hat{x}\left( k \right)$ can be updated by a gain learning rate $K\left( {k + 1} \right)$ as follows:

10 $$ \hat{x}\left( {k + 1} \right) = \hat{x}\left( k \right) + K\left( {k + 1} \right)\left\{ {y\left( {k + 1} \right) - \hat{x}\left( {k + 1} \right)} \right\} $$

where $K\left( {k + 1} \right)$ is an adjustable gain rate. It can be obtained through the incremental change of the state function and the measurement function.

According to the calculation of covariance, the state covariance can be updated finally to zero, i.e.,

11 $$ P\left( {k + 1} \right) = \left[ {I - K\left( {k + 1} \right)\frac{\partial h}{{\partial \hat{x}\left( {k + 1} \right)}}} \right]P\left( k \right) $$

where $I$ is a unit matrix, $\frac{\partial f}{{\partial \hat{x}\left( k \right)}}$ and $\frac{\partial h}{{\partial \hat{x}\left( k \right)}}$ are a partial derivative matrix, respectively.

When the initial filter value $\hat{x}\left( 0 \right)$ and the initial filtering covariance matrix $P\left( 0 \right)$ are acquired, the recursive algorithm is calculated in the following procedure.

$\hat{x}\left( 0 \right)$, $P\left( 0 \right)$ → $P\left( 1 \right)$ and $\hat{x}\left( 1 \right)$ → $K\left( 1 \right)$ and $\hat{x}\left( 1 \right)$ → $\hat{x}\left( 2 \right)$ and $P\left( 2 \right)$ → ……, which can be calculated step by step to estimate the system state. Obviously, the recursive equations all have to be implemented online.

In order to make the prediction model more accurate in application, the state of the above traffic flow model is constantly updated by using the interaction of states of each section. It is necessary to calculate the corresponding partial derivative matrix in the recursive equation and obtain the expression consistent with the above recursive Eq. (11), so that online recursive calculation can be carried out to obtain the estimated value of system state at each moment.

If the probability update of state transitions is assumed as $\mu_{k}^{(i)}$, then the final prediction of state interactive update is:

12 $$   \mathop x\limits^{\Lambda } _{{k\left| k \right.}}  = \sum\limits_{i} {\mathop {x_{{k\left| k \right.}} }\limits^{{\Lambda ^{{(i)}} }} \mu _{k}^{{(i)}} }   $$

From the definition of variance and mean, the total covariance of corresponding state error is updated as:

13 $$  P_{{k\left| k \right.}}  = \sum\limits_{i} [ P_{{k\left| k \right.}}^{{(i)}}  + E(\mathop x\limits^{\Lambda } _{{k\left| k \right.}}  - \mathop {x_{{k\left| k \right.}} }\limits^{{\Lambda ^{{(i)}} }} )(\mathop x\limits^{\Lambda } _{{k\left| k \right.}}  - \mathop {x_{{k\left| k \right.}} }\limits^{{\Lambda ^{{(i)}} }} )^{\prime}]\mu _{k}^{{(i)}}  $$

### Application of model

In here, an interval with an intersection is discussed. For each road in the intersection, only three equal length sections are discussed: one entrance, one driving section, and one exit.

Only the entrance section of the first road section and the exit section of the third road section provide detection data of vehicle flow and average speed as the input and output measurement values of the state estimation model. Take the flow at the head of the first section $q_{0}$, the average velocity $v_{0}$ as the input value. Take the density at the end of the third section $\rho_{3}$, the average velocity $v_{3}$ as the output. The purpose of state estimation is to accurately estimate the value of traffic variable in the section without detector according to the measured value of detector through constant state updating operation. During the experiment, it is assumed that the initial state value is the given normal value.

According to the model equation based on^1,2^, the traffic volume of each section is expressed by the density and speed $\rho_{i} \left( k \right)$ and $v_{i} \left( k \right)$ at three-way intersection, where $i = 1,2,3$.

### Comparison between iterative update and existing algorithm

1. Initial conditions

Simulate the above models. Assume the traffic density is uneven that is caused by sudden traffic accidents on the first road segment. The simulation parameters in the model are based on the values in^2^, i.e., $v_{f}$ = 60 (km /h), $\rho_{cr}$ = 20 (veh/m), $b$ = 3, $\tau$ = 19.4/3600, $\gamma$ = 34.7, $\xi$ = 1, $\lambda$ = 1, $\alpha$ = 0.95, $\Delta$ = 0.8 km.

Give initial state vector is: $$x_{0} = \left[ {\begin{array}{*{20}c} {10} & {10} & {10} & 0 & 0 & 0 \\ \end{array} } \right]^{\prime }$$,

Give covariance matrix is: $$P_{0} = \left[ {\begin{array}{*{20}c} {10} & 0 & 0 & 0 & 0 & 0 \\ 0 & {10} & 0 & 0 & 0 & 0 \\ 0 & 0 & {10} & 0 & 0 & 0 \\ 0 & 0 & 0 & {0.1} & 0 & 0 \\ 0 & 0 & 0 & 0 & {0.1} & 0 \\ 0 & 0 & 0 & 0 & 0 & {0.1} \\ \end{array} } \right]$$.

Model probability of state transition is: $$\mu_{0} = \left[ {\begin{array}{*{20}c} {0.6} & {0.2} & {0.2} \\ \end{array} } \right]^{\prime }$$.

2. Comparison of simulation results of the two algorithms

**Figure 2:**
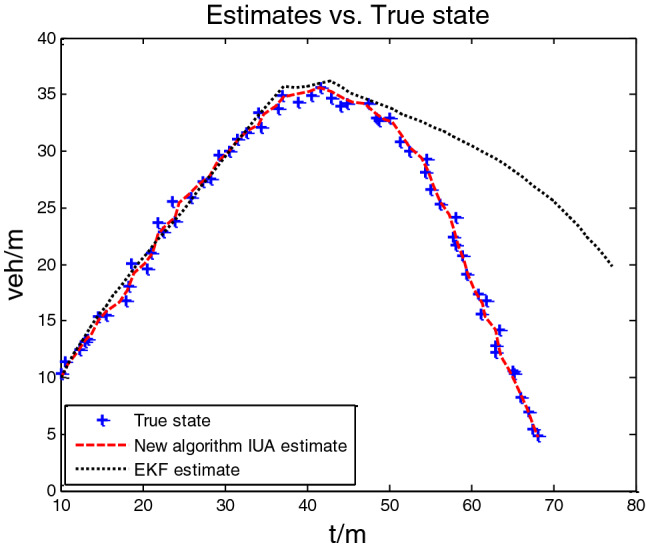
Comparison of estimation of IUA and EKF algorithms for traffic flow density.

**Figure 3:**
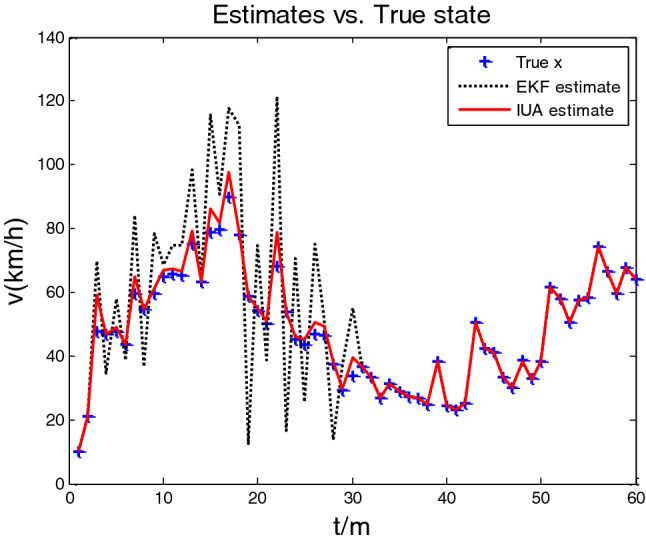
Comparison of estimation of IUA and EKF algorithms for traffic flow speed.

Using the iterative update algorithm (IUA) proposed in this paper and the existing EKF algorithm^19^, compare the results of the estimation of traffic density and traffic speed on the three-section viaduct highway within 90 s of simulation, as shown in Figs. 2 and 3. Figures 2 and 3 show the estimation of density and speed on traffic flow by IUA and EKF algorithms, respectively, which a predicted value is relative to the true value. From two figures, the estimation of IUA algorithm is better than that of EKF algorithm. IUA estimation curve basically coincides with the actual truth curve, while the EKF algorithm is relatively poor.

In addition, to evaluate the accuracy of the proposed IUA algorithm, it can be defined as follows:

Assume the total number of experiment statistics is $N$, the number $n$ of correct prediction is counted by the counting program, then the probability prediction $P$ of the precision is defined as

14 $$ P = \frac{n}{N} $$

**Figure 4:**
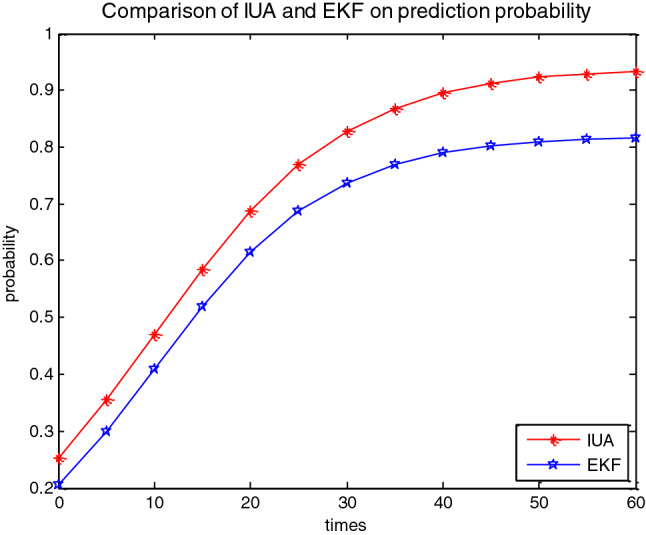
Comparison of IUA and EKF algorithms on prediction probability of traffic flow.

For 200 statistical experiments, repeat 60 times, and use the counting program to count the effective responses $n$ at each times. Then, the probability precision is calculated by formulas (14), as shown in Fig. 4.

Compare the accuracy of the IUA algorithm with the existing better EKF algorithm, the experimental results show that the accuracy of the IUA algorithm is 92.36%, and that of EKF algorithm is 78.82%, as shown in Fig. 4 and Table 1. In addition, the prediction speed of IUA algorithm is 2.08 ms. However, that of EKF is 4.19 ms.

**Table 1:** Comparison of proposed IUA and existing EKF algorithms.

Prediction algorithms	Prediction accuracy (%)	Prediction speed (ms)	Anti-interference ability
IUA algorithm proposed	92.36	2.08	Strong
EKF algorithm	78.82	4.19	Weak

From Fig. 4 and Table 1, the correct prediction probability of IUA algorithm proposed in this paper is higher than that of the existing EKF algorithm, and the running time is smaller. By comparison, it is found that the interactive algorithm proposed in this paper has more accurate prediction estimate, faster prediction speed, stronger anti-interference, better effect of congestion evacuation, and solves the problem of road congestion. The method proposed in this paper not only greatly alleviates the traffic pressure, but also maximizes the complementary advantages between intersections, thereby balancing the cooperation between each intersection.

## Intelligent decision

Based on the video of the current moment and the decision-making system, drivers are informed of the possible highway condition, a smooth route near the driving expressways, and even the smooth exit and entrance to a clear highway. In the design of decision system of highway traffic, the traffic state of each section of the highway must be used as feedback information to determine the control quantity such as speed limit, entrance regulation rate and information release. Here is a new approach to intelligent decision making called inferential decision-making.

### Decision rules

Let the traffic condition set be $X = \left\{ {x_{1} ,x_{2} , \cdots x_{n} } \right\}$, decision sets $Y = \left\{ {y_{1} ,y_{2} , \cdots y_{m} } \right\}$, so a mapping $R$ from $X$ to $Y$ is an optimal decision rule. For example, if $x_{i}$ is clear of traffic, then decision $y_{j}$ is $d_{ij}$ to proceed; or if $x_{i}$ is traffic congestion, then decision $y_{j}$ is to change the degree of driving on the current expressway road to $d_{ij}$, and so on. Where $d_{ij} = \mu_{X} \left( {x_{i} } \right) \wedge \mu_{Y} \left( {y_{j} } \right)$,$\mu_{X} \left( {x_{i} } \right)$ is the probability that $x_{i}$ is a member of a given set of conditions $X$_,_$\mu_{Y} \left( {y_{j} } \right)$ is the probability that $y_{j}$ is a member of the given set of states $Y$. Then, this decision rule is described as follows:

If a condition $x$ is $A$, then decision $y$ is $B$",

If $x$ is $A^{\prime}$, then decision $y$ is $B^{\prime}$.

It can be defined as $B^{\prime} = A^{\prime} \circ \left( {A \to B} \right)$. That is, conclusion $B^{\prime}$ can be obtained by combining $A^{\prime}$ with the inference relation from $A$ to $B$.Here, $A$ is the condition set of the state, and $B$ is the decision set based on condition $A$. The above rules can be simply described as follows:

It is known that when $A$ and $B$, the output is $C$, that is, there are inference rules.

IF $A$ AND $B$, THEN $C$.

To find what the decision output $C^{\prime}$ should be when $A^{\prime}$ and $B^{\prime}$.

### Establishment of decision model

1. Basic symbol and definition

To establish the regional traffic decision model, the following symbols and definitions are introduced:

(i) $Q_{i} \left( t \right)$ denotes the vector of the number of vehicles waiting at intersection $i$ at time $t$. $Q_{i} \left( t \right) = \left[ {Q_{i,E} \left( t \right),\;Q_{i,S} \left( t \right),\;Q_{i,W} \left( t \right),\;Q_{i,N} \left( t \right)} \right]$. Where $Q_{i,E} \left( t \right),\;Q_{i,S} \left( t \right),\;Q_{i,W} \left( t \right),\;Q_{i,N} \left( t \right)$ represent the number of vehicles in the east, south, west and north directions waiting at intersection $i$ at time $t$, respectively.
(ii) $Q_{i}$ represents the vector of the threshold of vehicle number at the intersection $i$, $Q_{i} = \left[ {Q_{i,E} ,\;Q_{i,S} ,\;Q_{i,W} ,\;Q_{i,N} } \right]$, where $Q_{i,E} ,\;Q_{i,S} ,\;Q_{i,W} ,\;Q_{i,N}$ indicate the thresholds of the number of vehicles waiting in different directions, respectively, which can be modified according to the actual situation.
(iii) $S$ represents the set of all possible strategies or actions in the decision. All feasible strategies of a decision are a finite set, where all strategies are rule sets. Assume $S = \left\{ {s_{1} ,s_{2} , \cdots ,s_{n} } \right\}$, each $s_{i}$ is the following rule shown in “decision model”.

2. Decision model

$$s_{1}$$:
If $Q_{i,E} \left( t \right) > Q_{i,E}$ and $Q_{i,W} \left( t \right) > Q_{i,W}$, but $Q_{i,S} \left( t \right) < Q_{i,S}$ and $Q_{i,N} \left( t \right) < Q_{i,N}$, then go straight in east and west direction, after 10 s, make a double left turn until 30 s to satisfy the red light of the east and west turns direction.
$$s_{2}$$:
If $Q_{i,S} \left( t \right) > Q_{i,S}$ and $Q_{i,N} \left( t \right) > Q_{i,N}$, but $Q_{i,E} \left( t \right) < Q_{i,E}$ and $Q_{i,W} \left( t \right) < Q_{i,W}$, then go straight in north and south direction, after 10 s, make a double-left turn from north to south until 30 s to satisfy the red light in north–south left turn direction.
$$s_{3}$$:
If $Q_{i,E} \left( t \right) < Q_{i,E}$ and $Q_{i,W} \left( t \right) < Q_{i,W}$ and $Q_{i,N} \left( t \right) < Q_{i,N}$, but $Q_{i,S} \left( t \right) > Q_{i,S}$, then it is necessary to turn left to the north and northeast for traffic.
$$s_{4}$$:
If $Q_{i,E} \left( t \right) > Q_{i,E}$ and $Q_{i,W} \left( t \right) > Q_{i,W}$, but $Q_{i,S} \left( t \right) > Q_{i,S}$ and $Q_{i,N} \left( t \right) > Q_{i,N}$, at the same time, $\left| {Q_{i,E} \left( t \right) - Q_{i,E} } \right| > \left| {Q_{i,S} \left( t \right) - Q_{i,S} } \right|$ and $\left| {Q_{i,E} \left( t \right) - Q_{i,E} } \right| > \left| {Q_{i,N} \left( t \right) - Q_{i,N} } \right|$ and $\left| {Q_{i,W} \left( t \right) - Q_{i,W} } \right| > \left| {Q_{i,S} \left( t \right) - Q_{i,S} } \right|$ and $\left| {Q_{i,W} \left( t \right) - Q_{i,W} } \right| > \left| {Q_{i,N} \left( t \right) - Q_{i,N} } \right|$, then go straight in east and west direction, or north–south vehicles can choose a nearby intersection to divert to other unblocked expressway roads.
$$s_{5}$$:
If $Q_{i,E} \left( t \right) > Q_{i,E}$ and $Q_{i,W} \left( t \right) > Q_{i,W}$ and $Q_{i,N} \left( t \right) > Q_{i,N}$, but $Q_{i,S} \left( t \right) < Q_{i,S}$, simultaneously, $\left| {Q_{i,E} \left( t \right) - Q_{i,E} } \right| < \left| {Q_{i,N} \left( t \right) - Q_{i,N} } \right|$ and $\left| {Q_{i,W} \left( t \right) - Q_{i,W} } \right| < \left| {Q_{i,N} \left( t \right) - Q_{i,N} } \right|$ and $\left| {Q_{i,S} \left( t \right) - Q_{i,S} } \right| < \left| {Q_{i,N} \left( t \right) - Q_{i,N} } \right|$, then turn left to the south and southwest vehicles pass, or both east–west and north-bound vehicles can choose the nearby intersection to divert to other unblocked expressway roads.
  …

The above rules are common: since there are two cases of traffic flow and threshold in each direction, and the four directions are considered at the same time, there is $2^{4}$ total of six cases similar to $s_{1}$, $s_{2}$ and $s_{3}$. Then the difference between the traffic flow in each direction and the threshold should be considered in the remaining $2^{4} - 6 = 10$ cases. However, there are 4 differences. When each difference is compared with 3 differences in the other direction, 3 cases appear $3 \times 10 = 30$ differences are obtained. Then, there are $30 + 6 = 36$ cases altogether. Therefore 36 rules are defined and 36 elements are contained in $S$, that are $s_{i}$ $i = 1,2, \ldots ,36$.

### Decision coordination

At a certain intersection at a certain time, if a certain rule $s_{i}$ is better than any other rule to make the expressway road smoother that is, the dredging time of the intersection is shorter, then rule $s_{i}$ wins and is denoted as $s_{i}^{ * }$. The coordination model is the game algorithm as follows.

#### Coordination model

A game with $n$ rules is described as $G = \left\{ {S,\;U} \right\}$, where $S$ is the rule set and $U$ is the advantage obtained by comparing two rules. For example, the advantage is that the expressway roads are smoother, or the intersection is cleared in shorter time, or congestion is cleared, and so on. If $m$ advantages exist, set $U = \left\{ {u_{1} ,u_{2} , \ldots ,u_{m} } \right\}$. If strategy $s_{i}^{ * }$ in this problem is a Nash equilibrium, then it must satisfy

15 $$ u_{j} \left( {s_{i}^{ * } ,\;s_{{i^{c} }} } \right) \ge u_{j} \left( {s_{i} ,\;s_{{i^{c} }} } \right)\quad \quad \forall s_{i} \in S $$

where, $s_{i}^{ * }$ represents the strategy chosen of the $i{\kern 1pt} {\text{th}}$ rule; $s_{{i^{c} }}$ represents the strategy of all rules except $i$; $u_{j}$ is the $j{\kern 1pt} {\text{th}}$ advantage. Or reorder by advantage, it can get a balance under Nash equilibrium.

#### Coordination algorithm

The whole traffic coordination process in the region is divided into three levels. The lower level is the coordination between the intersection and its adjacent intersection. The middle layer is the coordination between regional sections and intersections. The upper layer is the coordination between the area segment and its adjacent area segment.

**Figure 5:**
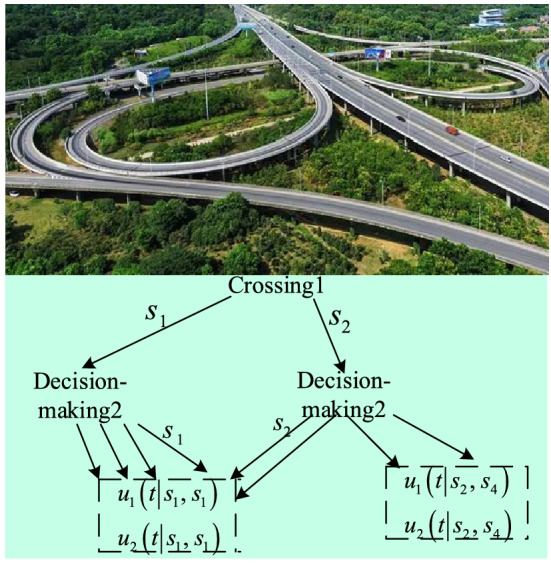
Game tree.
  The highways studied in here are not only urban roads and expressways, but also expressways outside the city, including mountain expressways. The intersections are in not only urban roads intersections, but also three intersections of expressways on-ramps and off-ramps, such as Fig. 5.
Step 3 If equilibrium exists, the action strategy of the decision is the strategy when equilibrium is achieved. Each decision controls the road mouthpiece according to this strategy, and the coordination ends. If there is no equilibrium, a request is made to the decision at that intersection.
Step 4 Regional decision making responds to the request, carries out game coordination for the intersection decision under its jurisdiction, and seeks the equilibrium point. If the equilibrium point does not exist, the regional decision making sends a request to the neighboring decision making.
Step 5 The decisions of adjacent regions respond to the request, carry out game coordination, and seek the equilibrium. If the equilibrium point does not exist, the coordination fails, then each decision keeps the original strategy unchanged.

Step 1 If the number of vehicles queuing at intersection 1 exceeds the threshold, a request is sent to adjacent intersection 2. In here, the threshold is obtained through a large number of congestion experiments, and then set by the algorithm program in the established model.
Step 2 Adjacent intersections respond to the request and build a game tree as shown in Fig. 5. The letters on the branches of the game tree represent the rule strategy, and the expressions in the block diagram represent the advantages of the rule comparison. Only three groups of advantage values are given in the figure, and other analogies are used to search the game tree and find the balance point according to Eq. (15).

**Figure 6:**
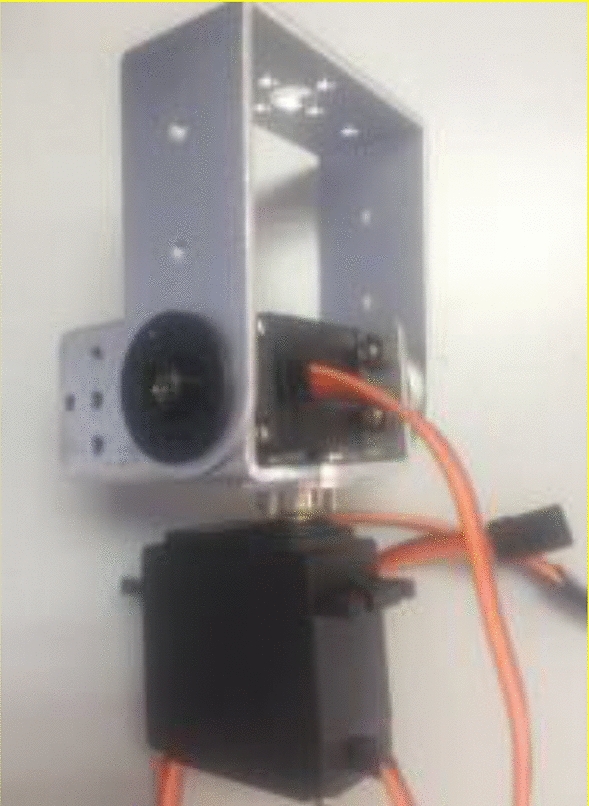
Developed hardware device.

**Figure 7:**
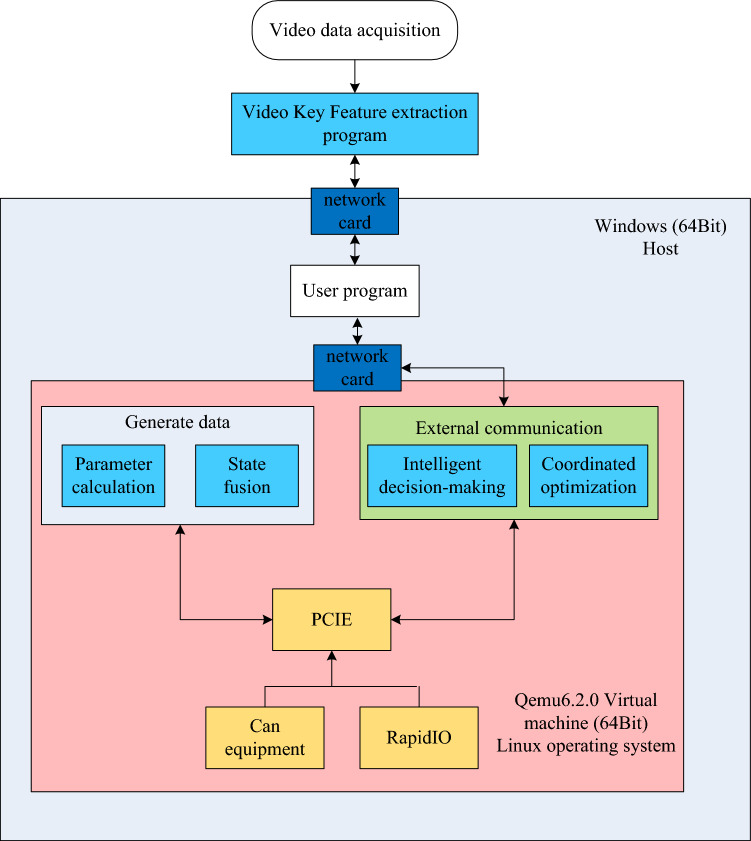
Developed traffic prediction and decision control model.

For this developed decision model, a hardware device is configured to provide navigation services for guidance vehicles on traffic congestion section, as shown in Fig. 6. Moreover, the software running environment is Matlab platform for proposed algorithms. Each communication interface protocol is written by Python. The hardware is constructed by using microelectronic components to develop an embedded system under Qemu equipment, as shown in Fig. 7.

### Comparison between proposed reasoning decision and existing methods

#### Calculation of comparison index

To evaluate the decision effect, the correct decision probability $P_{c}$ is to treat two discriminant results as the sole probability. Assume $N_{l} = \mathop {\max }\limits_{i} \left\{ {n_{il} } \right\}$, where $n_{il}$ is the times of all examined sections participating in matching test rules at time $l$, respectively. $N_{c}$ is the number of correct decisions. So there are

16 $$ P_{c} = \frac{{N_{c} }}{{N_{l} }} $$

Obviously, the above result is different for different $l$, but as $l$ increases,$P_{c}$ will approach a certain stable value.

#### Simulation results and analysis

**Figure 8:**
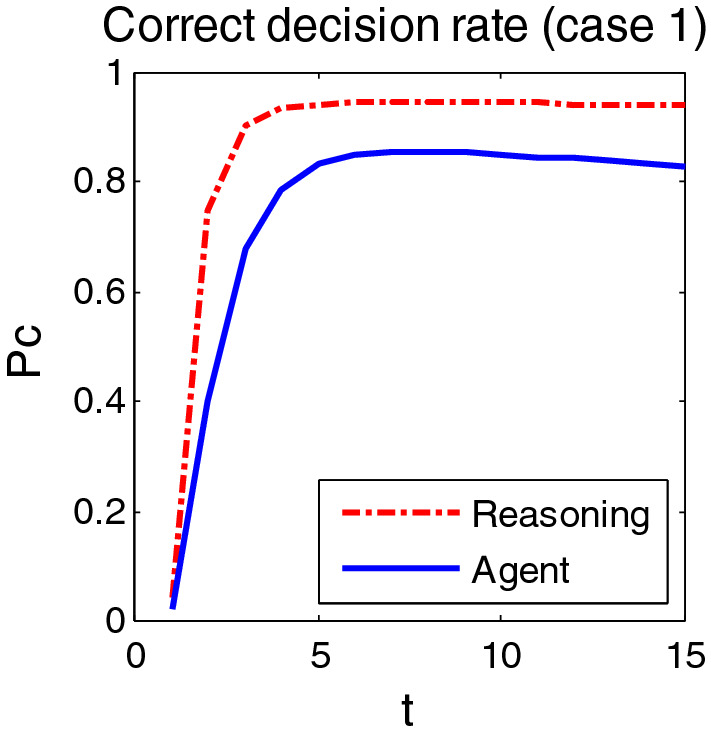
Correct decision rate in case 1.

**Figure 9:**
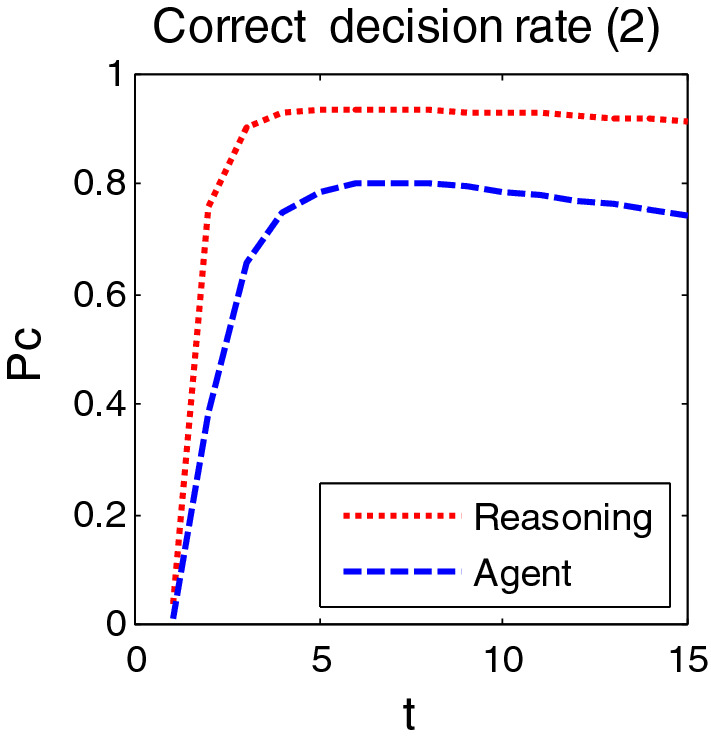
Correct decision rate in case 2.

The simulation was carried out in two cases. In case 1, the medium density traffic environment was simulated, and the target of entering the public area was 50 batches. Scenario 2 simulates the dense traffic environment, and the target of entering the public area is 100 batches. During the simulation, the random number seed was selected as 15, and the collection period was 4 s. Figures 8 and 9 show the correct decision rates at two cases 1 and 2 after 60 simulations, respectively.

#### Performance analysis

As can be seen from Figs. 8, 9, the correct decision rate in dense environment is slightly lower than that in medium density environment. In addition, the decision of reasoning in the overall performance is better than the existing Agent method, because it not only has good decision-making effect, fast processing speed, but also strong anti-noise ability. More than 90% accuracy of decision is achieved in the environment with dense target. In the medium density environment, the correct decision rate of the inference decision method is 91.28%, 8% higher than that of the Agent method. The processing speed is 0.0936 s, and the Agent method is 0.0780 s, both less than 1 s.

Compared with Agent, the biggest advantage of the reasoning decision method in this paper is that it can be applied not only to the dense target environment, but also to the larger system with model transformation and transmission error. But too many parameters of system and the complicated setting are its disadvantage. While Agent is good at universality and coordination, it is too sensitive and unsatisfactory in anti-noise ability. Another disadvantage is that once a node is changed, many other nodes will be affected.

#### Comprehensive comparison

To evaluate the comprehensive performance of each algorithm, a comprehensive comparison is conducted based on the correct decision rate, calculation speed, anti-noise, adaptability to environment and parameter setting requirements. With the quantitative and qualitative methods, the comparison evaluates the advantages and disadvantages of reasoning decision method and agent method. Table 2 shows the comprehensive comparison results.

**Table 2:** Comprehensive comparison between inference decision method and Agent method.

Algorithms	Mean of correct decision rate $\overline{P}_{c}$ (Case1)	Computing speed (Case1)	$\overline{P}_{c}$ (Case2)	Ability to anti- noise	Adapt to environment	Parameter Setting Requirements
Inference Decision	0. 9128	0.0936 s	0.9012	Strong	Dense target	Yes
Agent	0.8265	0.0780 s	0.7755	Weak	Sparse target	No

The correct average decision rate in Table 2 refers to the average of 15 time steps of each algorithm in the given simulation experiment environment 1 and environment 2 after 60 simulation experiments. They are, in fact, the spatially and temporally average of the rate of correct decisions, and therefore the overall average of the rate of correct decisions. The calculation speed is the calculation time used by the algorithm to calculate 15 time steps in the first simulation environment. It is only the calculation time of the algorithm itself. The computer used for simulation is Intel I7, 4-core, main frequency ≥ 2.2 GHz, 128 GB memory, and the programming language used is Matlab. The anti-noise capability in Table 2 is determined by adding some disturbance to the model parameters and some disturbance estimation to the sampling function of the algorithm. The adaptive environment in the table refers to the environment where the algorithm is applicable, and the parameter setting requirement refers to that the algorithm contains some parameters set according to the actual environment.

## Conclusion

This paper provides dynamic model state estimation from actual highway traffic data thus making it feasible to monitor traffic flow, which is extended to longer highway sections by dividing them into sections and calculating each dynamic parameter separately. These tests had been realized in practical traffic application on three-way intersection.

When the model parameters exceed 10 dimensions and there are more than two kinds of noise interference, the accuracy of the proposed model is not very good, and further optimization of the model design will be required in the future. At the same time, according to the actual control needs and the setting of sampling time, the number of state quantities will be determined by the maximum time required for each calculation of state under certain circumstances. Thus, the maximum number of expressways between two detectors on the highway can be roughly estimated, which will actually offer guidance on saving expenses in the process of transportation equipment installation, and cut down on consumption of resources.

## Data Availability

All data generated or analyzed during this study are included in this published article. We do wish to share our raw data, and these data are original.
